# Infection, immunity, and surveillance of COVID-19

**DOI:** 10.1371/journal.pmed.1004132

**Published:** 2022-11-10

**Authors:** Amitabh Bipin Suthar, Christopher Dye

**Affiliations:** 1 Centers for Disease Control and Prevention, Atlanta, United States of America; 2 Department of Biology, University of Oxford, Oxford, United Kingdom; PLOS: Public Library of Science, UNITED KINGDOM

## Abstract

Dr Amitabh Suthar and Dr Christopher Dye give their perspective on infection, immunity and surveillance of COVID-19.

Between January 2020 and September 2022, nearly 7 million Coronavirus Disease 2019 (COVID-19) deaths were counted worldwide while the total number of deaths associated with COVID-19, i.e., deaths directly and indirectly associated with the COVID-19 pandemic, was probably 2 to 4 times higher [1,2]. While the COVID-19 death toll has been a headline statistic in daily news bulletins throughout the pandemic, COVID-19 case surveillance—including case incidence, hospital admissions, and deaths—and virologic surveillance (Severe Acute Respiratory Syndrome Coronavirus 2 (SARS-CoV-2) test positivity rates) are used to guide the application of public health and social measures [3,4]. Although case surveillance may capture most COVID-19 cases admitted to hospital and most COVID-19 deaths, variable testing criteria and case definitions, limited access to diagnostics, and inconsistent reporting affect the completeness of COVID-19 cases reported. Therefore, additional methods for tracking SARS-CoV-2 and COVID-19 are needed.

## Serosurveillance

One of the additional measurement methods is serological surveillance (serosurveillance), in which the detection of specific antibodies signals exposure to SARS-CoV-2 infection among members of a selected population [5]. The virtue, in principle, of serology is that it records all exposures to infection whereas cases of COVID-19 illness are likely to be under-reported. The serological profile of a population indicates not simply the number of people infected, but who, where, and when. Risk factors for infection can be investigated by comparing exposures among infected and non-infected people. Repeated cross-sectional, seroprevalence surveys further allow calculations of the rate of spread of infection through a population. Moreover, given the underreporting associated with case surveillance, coupling hospitalizations and deaths with serosurveillance to calculate infection-hospitalization rates and infection-fatality rates may provide more reliable COVID-19 severity estimates than can be gleaned from case-hospitalization and case-fatality rates from case surveillance data [6]. WHO has recommended COVID-19 serosurveillance using a standardized methodology—the UNITY Protocol—since early 2020 [7]. To date, there has been no comprehensive synthesis of surveillance data using this approach. Now, in their new meta-analysis, Bergeri and colleagues included nearly 965 seroprevalence studies sampling 5,346,069 participants from 100 countries to present a composite picture of the temporal and spatial distribution of SARS-CoV-2 infection worldwide [8]. We provide our perspectives on this article and SARS-CoV-2 serosurveillance.

Some of their findings confirm expectations: They show how seroprevalence has risen during the pandemic, but with geographical variation. Other findings reinforce data from other sources: they describe the surge of infections due to the spread of novel SARS-CoV-2 variants in Africa (beta), Southeast Asia (delta), and in Europe and the Americas (omicron). They provide some evidence that stringent public health and social measures limited SARS-CoV-2 transmission, as reflected by lower seroprevalence rates. Their data also reinforce concerns about inequitable access to vaccines: Seroprevalence changes due to vaccination were more common in high-income countries while seroprevalence changes due to infection were more common in low- and middle income countries. The data also point to uneven access to health services and diagnostics because the ratio of infections to reported cases was high in resource-constrained regions of the world, particularly Africa. But the analysis by Bergeri and colleagues also poses questions about the current and future value of serosurveillance for SARS-CoV-2 and other emerging pathogens. We comment on 3.

The first concerns the precision of the serological assays used. At their core, accurate measures of seroprevalence depend on having antibody tests with high sensitivity and specificity. On sensitivity, Bergeri and colleagues found that seroprevalence was relatively low in children less than 10 years old. Perhaps children were less frequently exposed to infection; but low prevalence might also be explained by the milder infections experienced by children, which perhaps stimulated weaker antibody responses and more false negatives. Antibody titers also tend to be lower in asymptomatic cases, a proportion of which may never become positive during the course of infection [7]. Another challenge to serosurveillance is that infection can be confounded by vaccination. Bergeri and colleagues countered this by using antinucleocapsid (N) antibodies to measure infection in countries where vaccines using only spike (S) protein antigens, i.e., mRNA vaccines, where delivered. However, in many low- and middle-income countries inactivated vaccines, such as Sinovac’s CoronaVac, Sinopharm’s BBIBP-CorV, or Bharat Biotech’s BBV152 COVAXIN, are also delivered [9]. Inactivated vaccines elicit both anti-S and anti-N responses and therefore antinucleocapsid (N) antibodies would not differentiate between infection and vaccination. In these countries, seroprevalence measurements had to be adjusted using accessory data on the fraction of people vaccinated. Given the challenges of tracking vaccinations administered, this may have biased estimates.

Second, serosurveillance has limited utility in tracking rapidly spreading infections. Point seroprevalence is an aggregate between seroconversion and seroreversion [10]. For SARS-CoV-2, the median time from exposure to seroconversion is about 3 weeks; the time to reversion is about 25 weeks [11]. So serosurveillance captures neither recent infection nor past reversion (Bergeri and colleagues did not allow for reversion in their estimation of seroprevalence). In a rapidly growing epidemic with a doubling time of less than 1 week [12], seroprevalence lags far behind the spread of infection. In general, failing to allow for antibody dynamics will typically underestimate the cumulative prevalence of infection. In the extreme, if serological surveys are spaced too far apart, they could entirely miss explosive, short-lived outbreaks of disease (or waves of transmission).

Third, Bergeri and colleagues argue that anti-SARS-CoV-2 antibodies are highly predictive of immune protection, as stated in WHO guidelines [8]. However, the detection of antibody does not guarantee immunity, whether it be protection from SARS-CoV-2 infection or from COVID-19 illness and death, nor does the absence of antibody reliably indicate susceptibility to infection or disease. The relationship between antibody and protection against SARS-CoV-2 or COVID-19 requires quantitative calibration [13,14], recognizing that protection depends both on humoral (antibodies and memory B cells) and cellular immunity (T cells) [15]. The calibration is necessarily different for infection and disease, and no general rules yet exist. It is telling that just 6 (0.6%) of the serological studies described by Bergeri and colleagues were based on tests that detect neutralizing antibodies—the antibodies that are most closely linked to functional immunity.

## Future of serosurveillance

Bergeri and colleagues have shown how serosurveillance can help to characterize nearly 3 years of the COVID-19 pandemic. They do not discuss, either on technical grounds or with respect to the limited financial resources of many national health services, how to prioritize serological surveys alongside other key elements of disease surveillance systems and health system strengthening. While core surveillance systems serve priority objectives (Table 1), WHO gives serological surveys a limited role during COVID-19 outbreak investigations, tracking infection, and retrospectively measuring the attack rate or the size of an outbreak [3]. Furthermore, serosurveillance is not considered to be a source of information to guide public health and social measures [4]. As we learn how to safely live with SARS-CoV-2, the experience that lies behind nearly a thousand serological surveys will be valuable in updating WHO guidance on the role, requirements, and use of serosurveillance data for SARS-CoV-2 and future health emergencies. Those updated recommendations should inform the decision of whether and how to invest, as Bergeri and colleagues propose, in “a global system or network for targeted, multi-pathogen, high-quality, and standardized collaborative serosurveillance” to monitor COVID-19 and other emerging pathogens.

**Table 1:** WHO’s core surveillance systems for COVID-19 [3].

*Surveillance system*	*Objective*	*Examples of data use*	*Challenges*
Case surveillance	Monitor trends in diagnosed cases, hospital admissions, and deaths of COVID-19	-Situational awareness of morbidity, mortality -Characterize risk factors for severe disease and death -Guide application of public health and social measures	-Quality and completeness varies across countries -Data may lag true transmission trends due to symptom onset, test seeking behaviors, diagnostic availability, and reporting/informatic challenges
Environmental surveillance of wastewater	Serve as an early warning indication of transmission trends	-Increased virus in wastewater can serve as a leading indicator of transmission	-Sanitation access may be limited in many settings and lead to limited generalisability of results
Health system surveillance	Monitor health system capacity to treat COVID-19 case load (e.g., % of hospital and ICU beds that are vacant, oxygen supplies, supplies of essential diagnostics and medicines, and health of essential health cadres)	-Divert people requiring care to sites with available beds -Consider public health and social measures when health system is saturated with severe cases	-Private hospitals may not be part of national reporting system -Countries may not have real-time monitoring of commodities
Genomic surveillance	Monitor for new variants	-Understand which variant is predominant nationally and in different regions -Track emergence of new variants	-Sequencing capacity (laboratory and workforce) may not exist in all countries -Costly
